# Evaluating Darbepoetin Treatment Strategies for Managing Anaemia in Haemodialysis Patients: Effects on All‐Cause Mortality From a Target Trial Emulation Using Electronic Health Record Data

**DOI:** 10.1002/pds.70507

**Published:** 2026-10-18

**Authors:** Kate Birnie, Fergus J. Caskey, Yoav Ben‐Shlomo, Dorothea Nitsch, Anna Casula, Eleanor J. Murray, Jonathan A. C. Sterne

**Affiliations:** ^1^ Population Health Sciences, Bristol Medical School University of Bristol Bristol UK; ^2^ Renal Unit Southmead Hospital Bristol UK; ^3^ The National Institute for Health and Care Research Applied Research Collaboration West (NIHR ARC West) at University Hospitals Bristol and Weston NHS Foundation Trust Bristol UK; ^4^ Department of Noncommunicable Disease Epidemiology London School of Hygiene & Tropical Medicine London UK; ^5^ Department of Nephrology Royal Free London NHS Foundation Trust London UK; ^6^ UK Renal Registry Bristol UK; ^7^ Boston University, School of Public Health Boston Massachusetts USA; ^8^ NIHR Bristol Biomedical Research Centre Bristol UK

**Keywords:** anaemia, electronic health records, erythropoiesis stimulating agents, haemodialysis, target trial

## Abstract

**Purpose:**

Anaemia is a common complication of chronic kidney disease, often treated with erythropoiesis‐stimulating agents (ESAs), but the optimal haemoglobin (Hb) target remains uncertain. Using electronic health records, we emulated a target trial comparing different Hb targets in haemodialysis patients to estimate their effects on mortality.

**Methods:**

We used electronic health records from UK kidney centres reporting to the UK Renal Registry. Adults aged ≥ 18 years, receiving haemodialysis for ≥ 3 months, and without marked ESA resistance were eligible. Using the clone‐censor‐weight method, we emulated trials comparing Hb targets: (1) 105–125 versus 95–115 g/L and (2) 125–145 versus 95–115 g/L. Darbepoetin dosing followed a standardised protocol with predefined dose adjustments and a maximum dose of 150 μg/week. The outcome was all‐cause mortality over 8 months.

**Results:**

The study included 8628 patients from 10 centres between 2004 and 2016; 62% male, median age 66 years (IQR 52–76). At baseline, mean Hb was 97.7 g/L (SD 15.1), and 79% of patients were receiving darbepoetin. The hazard ratio for mortality was 0.92 (95% CI 0.75–1.12) for the 105–125 versus 95–115 g/L comparison, and 1.20 (95% CI 0.94–1.54) for the 125–145 versus 95–115 g/L comparison. Adherence to the highest Hb target was lower, limiting precision.

**Conclusions:**

Targeting an Hb range of 105–125 g/L under controlled ESA dosing was not associated with increased mortality compared with 95–115 g/L. Targeting Hb levels above 125 g/L may increase mortality risk, consistent with previous trial findings.

## Key Points

- The optimal haemoglobin target for anaemia treatment in haemodialysis patients treated with erythropoiesis‐stimulating agents remains uncertain.
- Using a target trial emulation based on routine clinical data, we compared haemoglobin targets commonly used in practice and those evaluated in earlier trials.
- Targeting a haemoglobin range of 105–125 g/L under controlled ESA dosing was not associated with increased mortality at 8 months compared with 95–115 g/L.
- Targeting haemoglobin levels above 125 g/L was associated with a higher estimated mortality risk.
- Haemodialysis patients may benefit from modestly higher Hb, under a dosing strategy that limits changes in dose and the maximum permissible dose of ESAs.

## Plain Language Summary

Anaemia, which means having low levels of haemoglobin in the blood, is common in people receiving dialysis for kidney disease. It is usually treated with medicines that help the body make red blood cells, together with iron therapy. However, doctors are unsure what haemoglobin level should be aimed for. Earlier clinical trials did not show clear benefits from aiming for higher levels and sometimes found increased risks, such as heart problems, so new trials are unlikely. We used existing health records to recreate, as closely as possible, what a clinical trial would examine. Using data from more than 8000 people receiving dialysis in the UK between 2004 and 2016, we compared different haemoglobin targets. Aiming for a moderate range (105–125 g/L) did not increase the risk of death compared with a lower range (95–115 g/L), while aiming for levels above 125 g/L may increase risk. These results suggest that carefully managed, modestly higher haemoglobin targets may be safe and could help improve symptoms for people on dialysis.

## 1 Introduction

Anaemia, defined by the World Health Organisation as haemoglobin (Hb) levels < 130 g/L in men and < 120 g/L in non‐pregnant women [1], leads to reduced quality of life [2], fatigue [3], decreased exercise capacity [4] and shortness of breath [5] in individuals with chronic kidney disease (CKD) and end‐stage kidney disease who require dialysis [6]. Erythropoiesis stimulating agents (ESAs), along with intravenous iron supplementation, are used to increase Hb levels, but the optimal level of Hb that should be targeted remains unknown. UK clinical guidelines recommend a target Hb of 100–120 g/L [7] and a 2026 update to the Kidney Disease: Improving Global Outcomes (KDIGO) guideline recommends targeting Hb levels below 115 g/L [8].

Randomized trials in patients with CKD [9, 10, 11] and end‐stage kidney disease [12] found either no change or an increase in the risk of cardiovascular events with higher Hb targets. However, reaching a target Hb of 120 g/L requires high doses of ESA in some individuals, and high ESA doses are associated with a higher risk of death in observational studies and post hoc analyses of randomized trials [13, 14, 15]. It has been proposed that high ESA doses can cause thrombosis [16] in the presence of relative iron deficiency, as well as arterial hypertension, endothelial activation, increased platelet reactivity, increased blood coagulability, and accelerated tumour growth, and inflammation [17, 18, 19, 20]. As a result, commonly used Hb targets have an upper limit of 120 g/L.

It is also possible that the higher risks associated with high ESA doses simply reflect that ESA resistance is a marker of other co‐morbidities (inflammation and cardiovascular disease). If so, individuals without ESA resistance may benefit from a higher Hb target than the one typically used. Because it is unlikely that new randomized trials will be conducted, this question needs to be addressed using observational data. We used UK electronic health record data to emulate a target trial comparing the effects of ESA treatment strategies on mortality in haemodialysis patients. The target ranges compared were (1) 105–125 g/L versus 95–115 g/L and (2) 125–145 g/L versus 95–115 g/L.

## 2 Methods

### 2.1 Data

The UK Renal Registry (UKRR) collects clinical and biochemical data for all UK adults receiving kidney replacement therapy [21]. We obtained a bespoke data extraction from selected UK kidney centres reporting to the UKRR between 2004 and 2016. Centres with incomplete recording of erythropoiesis‐stimulating agent (ESA) prescribing were excluded, in line with UKRR guidance [22] (for further details see Text A1). Analyses were restricted to patients prescribed darbepoetin, as dose equivalency with other ESAs was insufficiently comparable for pooled analyses.

### 2.2 Emulating a Target Trial

The target trial emulation and analysis methodology has been described in detail previously [23]. Patients were eligible if they were ≥ 18 years old and had been on haemodialysis for ≥ 3 months at one of the participating kidney centres and either were receiving darbepoetin or, if not, had a haemoglobin < 110 g/L. We excluded patients with evidence of marked ESA resistance (operationalised as receiving a high darbepoetin dose [≥ 120 μg/week]) and with low Hb [< 80 g/L] at baseline. The outcome was all‐cause mortality over 8 months.

We first compared Hb target range 105–125 g/L with range 95–115 g/L. In this framework, the ‘target Hb’ is a conceptual assignment reflecting an intended strategy, rather than an observed laboratory value. These ranges were chosen to fall slightly above and below the UK guideline range of 100–120 g/L [7] while reflecting the spectrum of Hb targets recommended or observed in international guidelines. These ranges intentionally overlap to reflect a modest change in Hb target. We also compared target range 125–145 g/L with range 95–115 g/L. The 125–145 g/L Hb range was chosen to align with near‐complete correction of anaemia, consistent with historical randomized trials in which Hb targets approached or exceeded 130 g/L [9, 10, 11, 12].

Darbepoetin dose adjustments followed a standardised protocol designed to reflect typical clinical practice. Decisions to modify doses were based on current and recent Hb levels, whether the dose had been changed in the previous month, and observed Hb response to prior dosing adjustments (Figure 1). Dose changes were allowed only within a predefined acceptable range, with a maximum dose of 150 μg/week permitted (Figure A1). Patients showing signs of ESA resistance requiring dose escalations were censored unless the dose remained within the allowed limits. Additionally, a grace period (within which changes should be made) of up to 1 month was incorporated to reflect delays in dose implementation. This grace period helped accommodate treatment variability and minimised censoring caused by minor deviations from the dosing rules.

**FIGURE 1:**
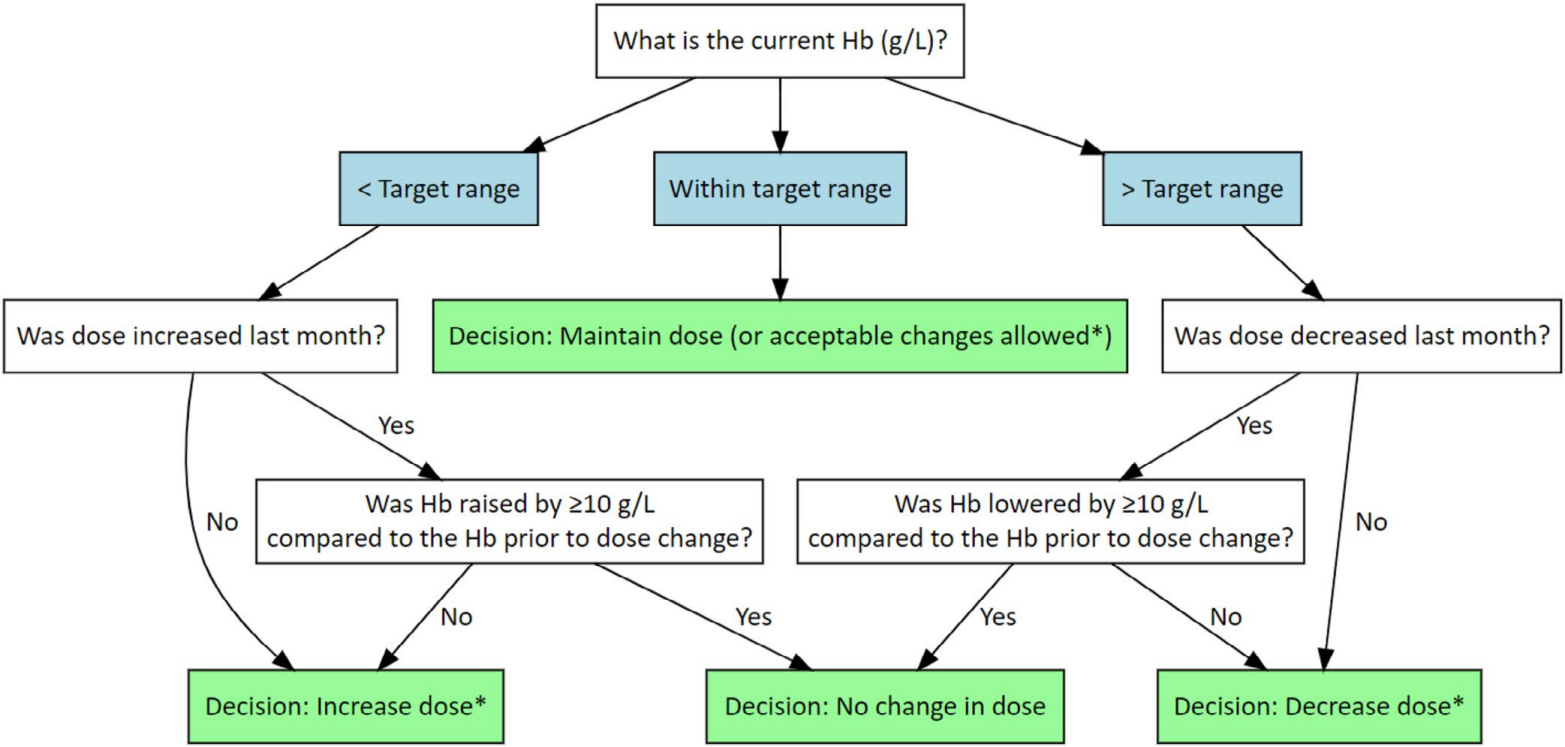
Dose decision protocol. *Note:* Hb: Haemoglobin. * When the decision is to change the dose, the new dose needs to be within acceptable levels (see Figure A1). The figure is conceptually based on Birnie et al. [23].

We used the three‐step ‘clone, censor, inverse‐probability weight’ framework for comparing sustained treatment strategies [24]. 1. Cloning: All patients met both strategies' eligibility criteria at baseline, defined as the latest of the dates when their dialysis centre became eligible and when they first met the eligibility criteria. At this point (time zero of the target trial), their follow‐up was duplicated, with one copy (‘clone’) assigned to each strategy being compared. 2. Censoring: Follow‐up ended when the patient's data was no longer compatible with the strategy assigned to the clone because they received a darbepoetin dose outside the acceptable range for the assigned strategy. 3. Inverse probability (IP) weighting, to account for the selection bias introduced by the informative censoring in the previous step.

Data were organised into discrete 28‐day intervals [25] for each patient. This interval length was chosen to reflect the timescale over which haemoglobin levels and ESA therapy are commonly reassessed in haemodialysis anaemia management [8]. Time zero was set at baseline. Each month, darbepoetin dose adjustments were recorded along with the corresponding Hb level that informed the treatment decision. Darbepoetin dose was recorded as zero if the patient was not receiving darbepoetin or as a numerical value if prescribed. Non‐zero doses were log‐transformed for normality. Follow‐up ended 8 months after baseline, death, kidney transplantation or a change to peritoneal dialysis, loss to follow‐up, or administrative end of follow‐up (31 December 2016), whichever happened first. Loss to follow‐up was defined as cessation of recorded observations in the dataset without a recorded death or another censoring event. Models adjusted for baseline demographic and clinical characteristics (age, sex, kidney centre, primary kidney disease and comorbidities) together with time‐updated measures that informed anaemia management and mortality risk, including haemoglobin, darbepoetin dose and markers of inflammation, nutrition and dialysis adequacy. For further details see Text A2. Covariates were selected a priori based on clinical knowledge and their anticipated role in treatment decisions and mortality risk.

### 2.3 Statistical Analysis

#### 2.3.1 Deriving Weights

Because follow‐up for a clone was censored when patients received a darbepoetin dose outside the acceptable range for the strategy assigned to that clone, models for treatment (darbepoetin dose levels) were used to derive the probability of being censored, based on covariates up to and including the previous month. The treatment models (described in detail previously) [23] included (1) logistic regression to estimate the probability of receiving zero darbepoetin, with separate models for patients receiving and not receiving darbepoetin in the previous month, to model cessation of darbepoetin and remaining off darbepoetin respectively; (2) a multivariable heteroskedastic linear regression model for log darbepoetin dose among those with non‐zero dose; and (3) multinomial logistic regression for extreme doses (e.g., 2.5 or 150 μg/week) in that month. The multinomial models were for all possible categories of dose given the previous month. For example, from a dose of 2.5 μg/week, the possible categories are: zero dose, the same dose, an increase consistent with both strategies, an increase consistent with the higher of the Hb target strategies only, or an increase inconsistent with both strategies. Due to small sample sizes in some categories, only Hb and month were included as covariates in the multinomial models.

The probability of adhering to the assigned treatment strategy in each month was derived by combining probabilities from all relevant models. The role of each component model and the patient populations to which it was applied are summarised in Table A1. The cumulative probability of remaining uncensored was derived by multiplying the probabilities of remaining uncensored during each month since baseline, with inverse probability of censoring weights (IPCW) derived as the reciprocal of these probabilities. Patients could also be censored for other reasons: separate logistic regression models, based on covariates up to and including the previous month, were used to calculate censoring weights for patients who changed from haemodialysis to peritoneal dialysis, received a kidney transplant or were lost to follow‐up and IPCW defined in the same way. IPCWs for each type of censoring were multiplied together to give the final consolidated weight for each strategy, patient and month.

Pooled logistic regression models, weighted using the IPCW for each strategy, were used to estimate the mortality hazard ratio (HR) for each comparison. Sensitivity analyses assessed the impact of large weights, and weighted survival curves were estimated for each strategy. To assess whether inverse probability weighting achieved balance across treatment strategies, we examined the distributions of key baseline and time‐updated covariates before and after weighting during follow‐up. Analyses were conducted in Stata 17 and R, and additional methodological details are shown in Text A3.

## 3 Results

### 3.1 Characteristics of the Study Cohort

A total of 8628 patients from 10 kidney centres were eligible for the study (Figure A2). All patients met eligibility criteria for each Hb target strategy at baseline, and subsequent comparisons were conducted using cloned cohorts to compare one target Hb range strategy to another. The median age was 66 years (IQR 52–76) and 5351 patients (62%) were male (Table 1). Diabetes and glomerulonephritis were the primary kidney diseases in 1783 (20.7%) and 1228 (14.2%) patients respectively. At baseline 6773 (78.5%) patients were being treated with darbepoetin, with a median dose of 30 μg/week (IQR 20, 50) and a mean Hb of 97.7 g/L (SD 15.1).

**TABLE 1:** Characteristics of study participants at baseline.

Covariate	Category/unit	*N* (%) or summary
Age (years)	Median (IQR)	66 (52–76)
Sex	Male	5351 (62.0%)
	Female	3277 (38.0%)
Primary kidney disease	Diabetes	1783 (20.7%)
	Glomerulonephritis	1228 (14.2%)
	Polycystic kidneys	534 (6.2%)
	Pyelonephritis	717 (8.3%)
	Other/uncertain/missing^a^	4366 (50.6%)
Year of entering study	2004	777 (9.0%)
	2005	737 (8.5%)
	2006	635 (7.4%)
	2007	593 (6.9%)
	2008	595 (6.9%)
	2009	570 (6.6%)
	2010	600 (7.0%)
	2011	1061 (12.3%)
	2012	777 (9.0%)
	2013	767 (8.9%)
	2014	640 (7.4%)
	2015	447 (5.2%)
	2016	429 (5.0%)
On darbepoetin treatment	Yes	6773 (78.5%)
	No	1855 (21.5%)
Darbepoetin dose, for those treatment (μg/week)	Median (IQR)	30 (20, 50)
Hb (g/L)	Mean (SD)	97.7 (15.1)
Ferritin (μg/L)	< 300	3018 (35.0%)
	300–449	1545 (17.9%)
	500+	1501 (17.4%)
	Missing	2564 (29.7%)
C‐reactive protein (mg/L)	0 or not tested	4432 (51.4%)
	0.1–4.9	426 (4.9%)
	5–19.9	1844 (21.4%)
	20+	1926 (22.3%)
Blood tests in previous month (N)	0	2072 (24.0%)
	1	3933 (45.6%)
	2	853 (9.9%)
	3	433 (5.0%)
	4+	1337 (15.5%)
Albumin (g/L)	< 35	4066 (47.1%)
	35–39	2497 (28.9%)
	40+	1750 (20.3%)
	Missing	315 (3.7%)
White blood count (10^9^/L)	< 6	2316 (26.8%)
	6–6.9	1380 (16.0%)
	7–7.9	1370 (15.9%)
	8–8.9	1113 (12.9%)
	9+	2449 (28.4%)
Adjusted calcium (mg/dL)	< 2.3	3667 (42.5%)
	2.3–2.39	1741 (20.2%)
	2.4–2.49	1383 (16.0%)
	2.5+	1428 (16.6%)
	Missing	409 (4.7%)
Urea reduction ratio (%)	< 60	1692 (19.6%)
	60–69	1826 (21.2%)
	70–74	994 (11.5%)
	75–79	633 (7.3%)
	80+	320 (3.7%)
	Missing	3163 (36.7%)

^a^ Includes patients with hypertension, renovascular disease, other renal diagnoses, uncertain primary kidney disease, or missing data.

### 3.2 Treatment Patterns Captured by the Models

The treatment models used to derive the inverse probability weights were consistent with clinical expectations, with prior dosing history and recent haemoglobin trends influencing treatment decisions (see Text A4).

### 3.3 Comparison of Haemoglobin Target Ranges: 105–125 g/L vs. 95–115 g/L

For the comparison of haemoglobin target ranges 105–125 g/L versus 95–115 g/L, Hb levels started to diverge by the second month, with the 105–125 g/L target group maintaining higher Hb levels for the remainder of follow‐up (Figure 2, panel A). By month 5, mean Hb in the 105–125 g/L strategy was 114.9 g/L (95% confidence interval [CI]: 114.5, 115.4 g/L), while in the 95–115 g/L strategy, it was 111.5 g/L (111.1, 112.0 g/L). By month 5, the geometric mean darbepoetin dose (for those being treated with darbepoetin) was 39.3 μg/week (95% CI 38.4, 40.2 μg/week) in the 105–125 g/L strategy and 34.9 μg/week (95% CI 34.1, 35.7) in the 95–115 g/L strategy (Figure 2, panel C). As expected, the number of patients in the analysis reduced over time as patients stopped adhering to the assigned strategies, died, were censored or were lost to follow‐up. At 4 months 56% of patients remained in the 105–125 g/L strategy and 60% in the 95–115 g/L strategy. By 8 months, 37% remained in the 105–125 g/L strategy and 35% in the 95–115 g/L strategy.

**FIGURE 2:**
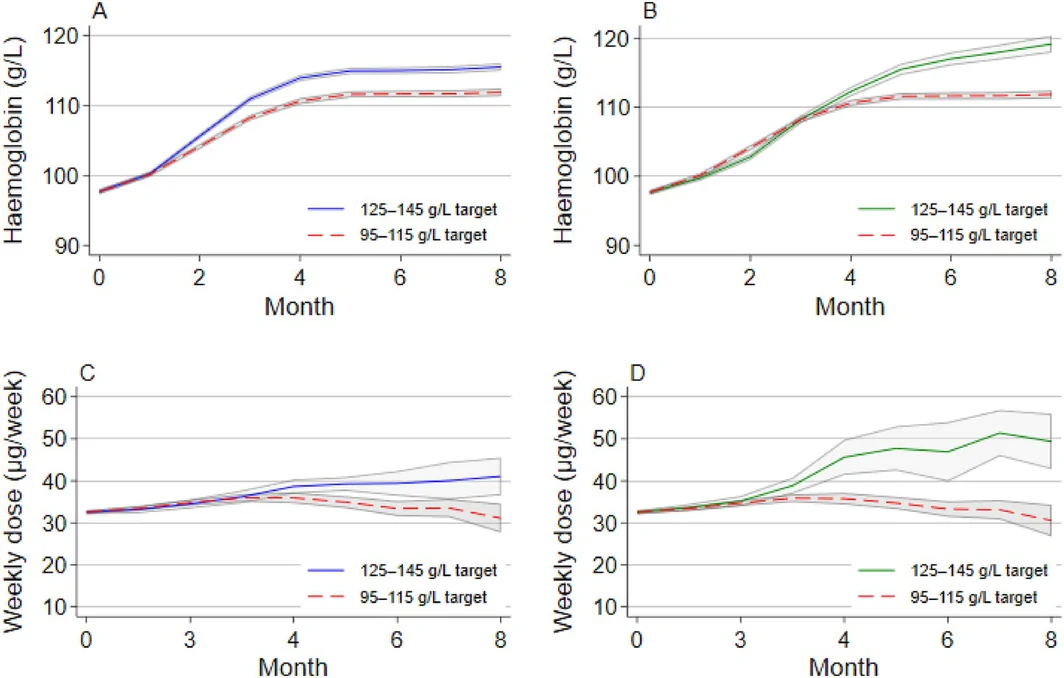
Mean Hb and geometric mean darbepoetin dose, with 95% CI, over time, by treatment strategy. Mean haemoglobin (Hb) with 95% confidence intervals from the unweighted analysis, shown to illustrate separation between treatment strategies. Geometric mean darbepoetin dose (for non‐zero doses) with 95% confidence (CI) intervals from a weighted analysis.

There were 373 deaths during 39 155 patient‐months follow‐up in the 105–125 g/L strategy group, compared to 434 deaths during 40 387 patient‐months in the 95–115 g/L strategy group. In the weighted analysis, which accounted for baseline and time‐updated covariates, the estimated hazard ratio (HR) comparing the 105–125 g/L versus the 95–115 g/L strategy was 0.92 (95% CI: 0.75, 1.12). The median weight was 1.5, with the 90th, 95th, and 99th percentiles at 5.2, 8.8, and 25.5, respectively. Truncating weights at the 99th, 95th, and 90th percentiles yielded similar effect estimates with narrower CIs: HRs of 0.91 (95% CI: 0.80, 1.03), 0.90 (95% CI: 0.82, 0.99), and 0.89 (95% CI: 0.82, 0.97), respectively. The unweighted estimate, which does not account for confounding, was 0.89 (95% CI 0.83, 0.94).

### 3.4 Comparison of Haemoglobin Target Ranges: 125–145 g/L vs. 95–115 g/L

For the 125–145 g/L Hb strategy, mean Hb increased across follow‐up, reaching 119.2 g/L (95% CI: 117.7, 120.6 g/L) by month 8 (Figure 2, panel B), when the geometric mean of darbepoetin dose (for those being treated with darbepoetin) was 49.4 μg/week (95% CI: 46.6, 52.3 μg/week) (Figure 2, panel D). There were 187 deaths during 21 663 patient‐months follow‐up in the 125–145 g/L strategy group. Only 667 patients (8%) in the 125–145 g/L strategy group remained uncensored by month eight, indicating that this strategy was not often followed in clinical practice. The median weight was 1.5, with the 90th, 95th, and 99th percentiles at 6.2, 11.2, and 43.8, respectively.

### 3.5 Sensitivity Analyses and Model Diagnostics

The estimated HR comparing the 125–145 g/L with the 95–115 g/L target was 1.20 (95% CI: 0.94, 1.54) after truncating the weights at the 99th percentile. Truncating weights at the 95th and 90th percentiles progressively attenuated effect estimates and narrowed CIs: HR 1.11 (95% CI: 0.93, 1.32) and 1.03 (95% CI: 0.88, 1.21), respectively. The fully weighted model produced unstable estimates due to a small number of very large weights and is therefore not presented. The unweighted estimate was 0.95 (95% CI 0.85, 1.07). The estimated survival curves for all three strategies are shown in Figure 3. The curve for the 125–145 g/L target strategy lies below those for the other two target ranges from around month 4 onward, although the confidence intervals are wide. Confidence intervals widened over time, reflecting greater uncertainty due to the increased proportion of patients whose follow‐up was censored, reducing the amount of available data at later time points. Tables A2–A9 show that weighting resulted in more comparable distributions of measured covariates across haemoglobin target strategies. Weighted distributions generally showed smaller differences between strategies than unweighted distributions. Some minor differences remained for some variables at later follow‐up, particularly for time‐updated inflammatory and nutritional markers (C‐reactive protein and serum albumin), when fewer individuals remained under observation.

**FIGURE 3:**
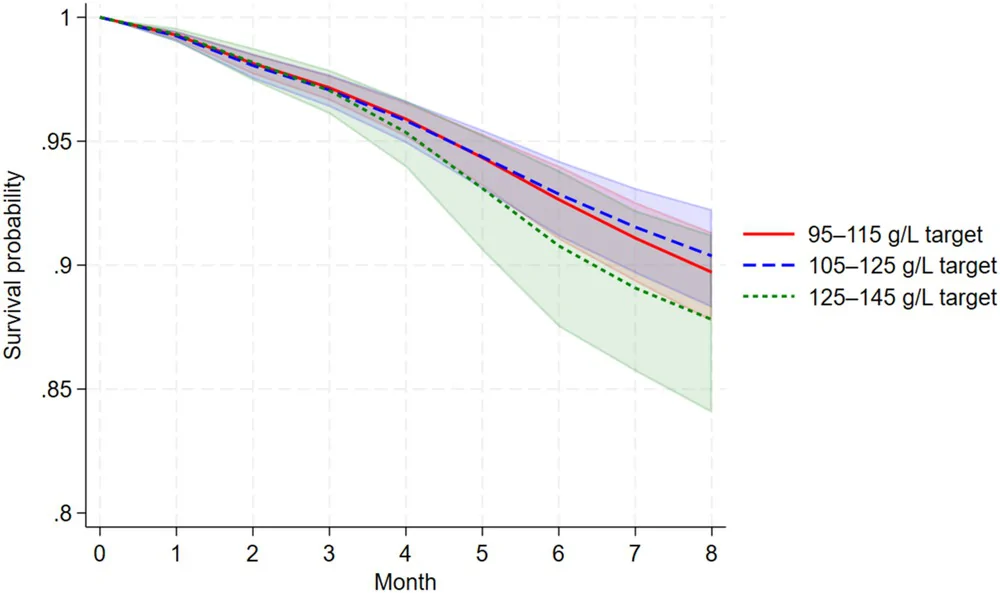
Estimated weighted survival curves for all‐cause mortality for the three target Hb strategies. *Note:* Shaded areas represent 95% confidence intervals derived from bootstrap resampling.

## 4 Discussion

Using observational electronic health record data from the UKRR, we emulated trials to evaluate the effects of different Hb target strategies on mortality among haemodialysis patients. Because these strategies cannot be distinguished by patients' characteristics at the start of follow‐up, we employed the clone‐censor‐weight method. We found no evidence of increased all‐cause mortality comparing the 105–125 g/L Hb with the 95–115 g/L target range over 8 months of follow‐up. The 105–125 g/L target is slightly higher than the UK Kidney Association Clinical Practice Guidelines recommendation (100–120 g/L [7]) and exceeds the KDIGO upper limit of 115 g/L [8]. In contrast, targeting 125–145 g/L was associated with a potential increase in mortality compared with 95–115 g/L.

Evidence from randomized trials has raised concerns about higher Hb targets. A randomized trial of 1233 haemodialysis patients with cardiovascular comorbidity, published in 1998 [12], was terminated early when it became clear that benefit from full correction of anaemia (compared to partial correction) was highly improbable. The CHOIR trial (published 2006) [11] randomized 1432 patients with CKD not on kidney replacement therapy (KRT) to Hb targets of 135 g/L or 113 g/L. Patients assigned to the higher Hb target had an increased risk (HR 1.34; 95% CI: 1.03, 1.74) of the primary composite outcome (death, myocardial infarction, hospitalisation for heart failure, or stroke) compared to the lower Hb target. In contrast, the CREATE trial (published 2006) [9], randomized 603 patients with CKD not on KRT to a target Hb of 130–150 g/L or 105–115 g/L: the HR for cardiovascular events was 0.78 (95% CI: 0.53, 1.14). The TREAT trial, published in 2009 [10], included 4038 patients with diabetes, CKD, and anaemia. Participants were randomly assigned to achieve a haemoglobin level of approximately 130 g/L using darbepoetin alfa or placebo with rescue darbepoetin alfa if Hb < 90 g/L. The HR for the composite outcome of death or cardiovascular events was 1.05 (95% CI: 0.94, 1.17), but stroke incidence was higher in the darbepoetin alfa arm (HR 1.92; 95% CI: 1.38, 2.68).

Across trials, patients assigned to higher Hb targets typically received median ESA doses two to three times greater than those assigned to lower targets [20]. Secondary analyses of CHOIR suggests that both failure to achieve the target Hb and high ESA doses were associated with worse outcomes [15]. An observational study using the target trial approach in 22 474 dialysis patients aged ≥ 65 years with diabetes and cardiovascular disease from the United States Renal Data System (USRDS) evaluated clinical strategies for managing anaemia with epoetin therapy [26]. The study found similar 6‐month survival targeting a medium haematocrit range (34.5%–39.0%) and a lower haematocrit range (30.0%–34.5%), HR 0.98 (95% CI 0.78–1.24). A further analysis of USRDS data using the target trial approach with the parametric g‐formula found that, compared to a low‐haematocrit strategy (30%–33%), the estimated risk of death was 4.6% higher (95% CI: 4.4–4.9) under a high‐haematocrit strategy (36%–39%) and 1.8% higher (95% CI: 1.7–1.9) under a mid‐haematocrit strategy (33%–36%) [27]. This study used an 18‐month follow‐up and included patients with a history of congestive heart failure or ischaemic heart disease in the 2 years prior to study entry.

Anaemia management in UK haemodialysis patients changed considerably between 2005 and 2013 [28]. There was a decrease in ESA use, the average dose administered, and the achieved Hb levels in UK haemodialysis patients over time. These trends likely reflect evidence from randomized trials and updated guidelines, prompting a more conservative approach to anaemia management. The Proactive IV Iron Therapy in Haemodialysis Patients (PIVOTAL) trial, published after our data extraction period, is likely to have further influenced clinical practice by demonstrating that a proactive high‐dose intravenous iron regimen reduced ESA requirements and improved clinical outcomes compared to a reactive low‐dose approach [29]. As a result, current ESA prescribing is often combined with diverse iron strategies, which can complicate the interpretation of ESA effects alone. By analysing data that precede the PIVOTAL trial, our study offers a more isolated assessment of ESA treatment strategies, minimising influence from evolving iron management practices. Our findings suggest that a modestly higher Hb target may not be detrimental to patient survival, providing that excessive ESA dosing is avoided.

A strength of our analysis was the availability of detailed observational data, which allowed us to emulate a target trial and assess dynamic treatment strategies reflecting clinical practice. This approach aligns with recent recommendations from the International Society of Nephrology consensus meeting on advancing clinical research in kidney disease, which advocates for the use of innovative trial designs and real‐world data to improve evidence generation in nephrology [30]. The UKRR is a large and representative database allowing trends in clinical practice patterns to be captured [31]. By structuring the data into monthly time periods we were able to incorporate information on Hb levels that informed darbepoetin treatment decisions. The study design utilised variability in practitioners' usual darbepoetin dosing practices over time, assuming this variation was not systematically linked to other, unmeasured factors influencing mortality. However, our study was limited by incomplete data returns, as some kidney centres do not routinely record computerised data on ESA dose or drug type, necessitating restriction to centres with adequate reporting. We selected 8‐month mortality as the primary outcome because, in our target trial emulation, longer follow‐up periods resulted in high censoring of clones, particularly in the 125–145 g/L Hb target group. Extending the follow‐up further would have reduced the effective sample size and increased the risk of instability in the inverse‐probability weighting framework. We acknowledge that many randomized trials of anaemia treatment in CKD/ESKD have used 3‐year follow‐up periods, and our findings should be interpreted in the context of this shorter follow‐up. A key limitation of this study is its observational nature: we cannot exclude unmeasured confounding. However, we controlled for the key variables that predict treatment decisions and/or are associated with all‐cause mortality. An additional consideration is that data were extracted from 2004 to 2016. This period encompassed publication of several influential ESA trials and subsequent changes to anaemia management guidelines, resulting in heterogeneity in haemoglobin targets and ESA dosing practices used by clinicians over the time‐period [28]. While this variation is an advantage because it enabled comparison of alternative treatment strategies, treatment practices have evolved since 2016, including the emergence of newer therapies for anaemia management. Therefore, the generalisability of our findings should be considered in this context. A further consideration is that the target trial was designed in a prevalent haemodialysis population. Patients were required to have been on haemodialysis for at least 3 months because anaemia management, ESA requirements and haemoglobin levels can be unstable around dialysis initiation, making treatment strategies more difficult to define and emulate reliably. Consequently, our findings apply to patients who survived and remained on haemodialysis for at least 3 months and may not generalise to haemoglobin‐targeting strategies initiated at dialysis commencement.

In conclusion, our target trial emulation using EHR data found no evidence that targeting a Hb range of 105–125 g/L, compared with a 95–115 g/L range, with a maximum darbepoetin dose of 150 μg/week, increases all‐cause mortality at 8 months in haemodialysis patients. However, targeting a range of 125–145 g/L may increase mortality compared with a 95–115 g/L range. Haemodialysis patients may benefit from modestly higher Hb, in terms of symptoms, under a dosing strategy that limits changes in dose and maximum permissible dose of ESAs.

## Author Contributions

J.A.C.S., K.B., F.J.C., Y.B.‐S. and D.N. designed the study. F.J.C. and A.C. advised on the data. K.B. performed the analyses. J.A.C.S. and E.J.M. advised on the analyses. All authors contributed to interpreting results. K.B. wrote the initial paper draft and all other authors edited and provided feedback on drafts. All authors read and approved the final manuscript.

## Funding

Kate Birnie was supported by a Medical Research Council (MRC) UK fellowship (MR/K021699/1). Yoav Ben‐Shlomo was partly funded by National Institute for Health and Care Research Applied Research Collaboration West (NIHR ARC West) and University of Bristol. Jonathan AC Sterne was supported by the NIHR Bristol Biomedical Research Centre.

## Ethics Statement

The processing of UKRR data for research has been approved by the NRES Committee North East Newcastle and North Tyneside 1 Research Ethics Committee, reference 21/NE/0045.

## Consent

The UKRR has section 251 permissions to use data for research without individual patient consent.

## Conflicts of Interest

The authors declare no conflicts of interest.

## Supporting information


**Data S1:** Supporting Information.
**Text A1:** Further details on data.
**Text A2:** Further information on covariates.
**Text A3:** Further details on statistical models.
**Text A4:** Treatment patterns captured by the models.
**Figure A1:** Acceptable dose changes (μ/week).
**Figure A2:** Flow chart of patients included in the study.
**Table A1:** Models used to estimate monthly adherence probabilities.
**Table A2:** Distribution of age (years; mean) before and after inverse probability weighting, by haemoglobin target strategy and follow‐up month.
**Table A3:** Distribution of sex (%) before and after inverse probability weighting, by haemoglobin target strategy and follow‐up month.
**Table A4:** Distribution of primary kidney disease (%), before and after inverse probability weighting, by haemoglobin target strategy and follow‐up month.
**Table A5:** Distribution of heart failure comorbidity status (%), before and after inverse probability weighting, by haemoglobin target strategy and follow‐up month.
**Table A6:** Distribution of time‐updated ferritin (μg/L; categories %) before and after inverse probability weighting, by haemoglobin target strategy and follow‐up month.
**Table A7:** Distribution of time‐updated serum albumin (g/L; categories %) before and after inverse probability weighting, by haemoglobin target strategy and follow‐up month.
**Table A8:** Distribution of time‐updated white blood count (10^9^/L; categories %) before and after inverse probability weighting, by haemoglobin target strategy and follow‐up month.
**Table A9:** Distribution of time‐updated C‐reactive protein (mg/L; categories %) before and after inverse probability weighting, by haemoglobin target strategy and follow‐up month.

## Data Availability

The dataset is not available due to privacy restrictions. Researchers may apply for access to UK Renal Registry data through the UK Kidney Association. Information and application procedures are provided at https://www.ukkidney.org/audit‐research/how‐access‐data.
