# Supplementary material for: Evaluating Darbepoetin Treatment Strategies for Managing Anaemia in Haemodialysis Patients: Effects on All‐Cause Mortality From a Target Trial Emulation Using Electronic Health Record Data
Source: Pharmacoepidemiol Drug Saf. 2026 Oct 18;35(11):e70507. doi: 10.1002/pds.70507 (PMC13677213; doi:10.1002/pds.70507)
Supplement: Supplementary file 1 [file PDS-35-e70507-s001.docx]

**Evaluating darbepoetin treatment strategies for managing anaemia in haemodialysis patients: effects on all-cause mortality from a target trial emulation using electronic health record data**

**Supplementary information**

**Text A1 Further details on data**

The UK Renal Registry (UKRR) collects clinical and biochemical data for all UK patients aged ≥18 years receiving kidney replacement therapy (KRT). A bespoke data extraction was obtained for selected kidney centres reporting to the UKRR. Following UKRR guidance, in each quarter of a calendar year, centres reporting <60% of haemodialysis patients being treated with erythropoiesis-stimulating agents (ESAs) were considered to have incomplete data so were excluded. This threshold reflects the registry’s standard for identifying under-captured ESA use rather than true low utilization. Data were extracted from 2004 to 2016, though the contribution period varied by centre based on when prescribing data were sufficiently complete.

**Text A2 Further information on covariates**

Baseline covariates included: patient age (years), sex, kidney centre (the largest centre was chosen as the reference category), primary kidney disease (diabetes, glomerulonephritis, pyelonephritis, polycystic kidneys and ‘other’ [hypertension, kidney vascular disease, other, uncertain]) and co-morbidities present at the start of KRT (angina, angioplasty, claudication, chronic obstructive pulmonary disease, diabetes not causing kidney failure, ischaemic / neuropathic ulcers, liver disease, malignancy, previous myocardial infarction within last 3 months prior to starting KRT, previous myocardial infarction >3 months prior to start of KRT, previous coronary artery bypass graft or coronary angioplasty, amputation for peripheral vascular disease, symptomatic cerebrovascular disease, heart failure, and whether the patient was a smoker). Time-updated covariates included: Hb (g/L), darbepoetin dose (µg/week), white blood cell count (10^9^/L), albumin (g/L), ferritin (µg/L), adjusted calcium (mg/dL), C-reactive protein (mg/L), urea reduction ratio (dialysis adequacy %) number of blood tests in the previous 28 days, time since eligibility into the study (months). Continuous time-updated covariates were categorised for modelling purposes. For variables with missing values, an additional "missing" category was created. This approach allowed all eligible patients to contribute to the analysis without requiring complete covariate information at each time point. For white blood count, missing values were rare (0.04%) and were assigned to a valid data category rather than modelled separately.

**Text A3 Further details on statistical models**

Pooled logistic regression models, weighted using the IPCW for each strategy, was used to estimate the mortality hazard ratio (HR) for each comparison. These models included cubic splines with 2 knots for month (to allow for non-linear relationships) and used robust standard errors for clustering by patient. In sensitivity analyses the IPCW were truncated at the 99th, 95th and 90th percentiles (chosen a priori) to mitigate the impact of large weights. We estimated weighted survival curves under each strategy using inverse probability of treatment and censoring weights, truncated at the 99th percentile to improve stability. Statistical analyses were conducted in Stata 17. Confidence intervals were obtained using a nonparametric bootstrap procedure with 1,000 replicates. Figures of flow diagrams and dose change visualisations were generated in R (version 4.5.1) using the DiagrammeR, ggplot2, dplyr, and tibble packages.

**Text A4**  **Treatment patterns captured by the models**

The treatment models used to derive the inverse probability weights were consistent with clinical expectations. Prior dosing history was strongly associated with current dose, and recent rises in haemoglobin were associated with dose reductions or stopping darbepoetin. Among those already receiving darbepoetin, patients with heart failure or with frequent blood tests in the prior month were more likely to have darbepoetin stopped. Among those not receiving darbepoetin, higher ferritin and CRP levels were associated with a lower likelihood of remaining on zero dose, consistent with anaemia management decisions in the context of inflammation.

**Figure A1** **Acceptable dose changes (µ/week)**

**Figure A2 Flow chart of patients included in the study**

Notes

ESA: Erythropoiesis-Stimulating Agent

**Table A1 Models used to estimate monthly adherence probabilities**

| **Model** | **Outcome at month t** | **Population** | **Contribution to adherence probability** |
| --- | --- | --- | --- |
| Logistic 1 | Zero darbepoetin at t | Zero dose at t−1 | Probability of remaining off treatment |
| Logistic 2 | Zero darbepoetin at t | Non-zero dose at t−1 | Probability of treatment cessation |
| Heteroskedastic linear model | Non-zero dose at t | Non-zero dose at t | The probability of dose being in the acceptable range |
| Multinomial models | Extreme dose at t | Extreme dose at t | The probability of a suitable dose |
| Censoring models | Dialysis modality change, transplant, loss to follow-up | All patients at risk | Additional censoring weights multiplied with treatment weights |

**Table A2 Distribution of age (years; mean) before and after inverse probability weighting, by haemoglobin target strategy and follow-up month.**

|  |  | Unweighted |  |  | Weighted |  |
| --- | --- | --- | --- | --- | --- | --- |
| Month | 95–115 g/L | 105–125 g/L | 125–145 g/L | 95–115 g/L | 105–125 g/L | 125–145 g/L |
| 0 | 62.8 | 62.8 | 62.8 | 62.8 | 62.8 | 62.8 |
| 1 | 62.9 | 62.9 | 62.9 | 62.8 | 63.0 | 63.0 |
| 2 | 63.2 | 63.0 | 62.9 | 62.7 | 62.8 | 62.9 |
| 3 | 63.6 | 63.2 | 63.0 | 62.8 | 62.8 | 63.0 |
| 4 | 63.8 | 63.3 | 63.3 | 62.7 | 63.0 | 63.5 |
| 5 | 63.8 | 63.4 | 63.4 | 62.6 | 63.0 | 63.4 |
| 6 | 64.0 | 63.6 | 63.4 | 62.7 | 63.0 | 63.7 |
| 7 | 64.0 | 63.7 | 63.5 | 62.7 | 62.9 | 64.2 |
| 8 | 64.3 | 63.8 | 62.8 | 62.4 | 62.8 | 64.3 |

Notes: Covariate distributions were assessed using inverse probability weights truncated at the 99th percentile across Tables A2-A9

**Table A3 Distribution of sex (%) before and after inverse probability weighting, by haemoglobin target strategy and follow-up month.**

|  |  |  | Unweighted |  |  | Weighted |  |
| --- | --- | --- | --- | --- | --- | --- | --- |
| Month | Sex | 95–115 g/L | 105–125 g/L | 125–145 g/L | 95–115 g/L | 105–125 g/L | 125–145 g/L |
| 0 | Male | 61.5 | 61.5 | 61.5 | 61.5 | 61.5 | 61.5 |
|  | Female | 38.5 | 38.5 | 38.5 | 38.5 | 38.5 | 38.5 |
| 1 | Male | 61.5 | 61.6 | 61.4 | 61.6 | 61.1 | 60.7 |
|  | Female | 38.5 | 38.4 | 38.6 | 38.5 | 38.9 | 39.3 |
| 2 | Male | 61.4 | 61.3 | 61.4 | 61.5 | 61.3 | 60.9 |
|  | Female | 38.6 | 38.7 | 38.6 | 38.5 | 38.7 | 39.2 |
| 3 | Male | 61.4 | 61.7 | 61.3 | 61.2 | 60.7 | 60.5 |
|  | Female | 38.6 | 38.4 | 38.7 | 38.8 | 39.3 | 39.5 |
| 4 | Male | 61.5 | 61.9 | 61.4 | 61.5 | 61.3 | 60.5 |
|  | Female | 38.5 | 38.1 | 38.6 | 38.5 | 38.7 | 39.5 |
| 5 | Male | 61.6 | 62.4 | 61.4 | 61.7 | 62.1 | 58.6 |
|  | Female | 38.4 | 37.6 | 38.6 | 38.3 | 37.9 | 41.4 |
| 6 | Male | 61.8 | 62.9 | 61.0 | 61.5 | 63.0 | 56.9 |
|  | Female | 38.2 | 37.1 | 39.0 | 38.5 | 37.0 | 43.1 |
| 7 | Male | 61.9 | 63.6 | 61.6 | 61.2 | 63.5 | 57.5 |
|  | Female | 38.1 | 36.4 | 38.4 | 38.8 | 36.5 | 42.5 |
| 8 | Male | 61.7 | 64.0 | 62.6 | 61.4 | 64.1 | 59.5 |
|  | Female | 38.3 | 36.0 | 37.4 | 38.6 | 35.9 | 40.5 |

**Table A4 Distribution of primary kidney disease (%), before and after inverse probability weighting, by haemoglobin target strategy and follow-up month.**

|  | Primary kidney disease |  | Unweighted |  |  | Weighted |  |
| --- | --- | --- | --- | --- | --- | --- | --- |
| Month |  | 95–115 g/L | 105–125 g/L | 125–145 g/L | 95–115 g/L | 105–125 g/L | 125–145 g/L |
| 0 | Diab | 20.6 | 20.6 | 20.6 | 20.6 | 20.6 | 20.6 |
|  | Glom | 14.3 | 14.3 | 14.3 | 14.3 | 14.3 | 14.3 |
|  | Polyc | 6.2 | 6.2 | 6.2 | 6.2 | 6.2 | 6.2 |
|  | Pyelo | 8.3 | 8.3 | 8.3 | 8.3 | 8.3 | 8.3 |
|  | Other | 50.5 | 50.5 | 50.5 | 50.5 | 50.5 | 50.5 |
| 1 | Diab | 20.6 | 20.6 | 20.6 | 20.4 | 20.6 | 20.5 |
|  | Glom | 14.2 | 14.2 | 14.1 | 14.3 | 13.9 | 13.9 |
|  | Polyc | 6.3 | 6.3 | 6.3 | 6.2 | 6.3 | 6.5 |
|  | Pyelo | 8.4 | 8.4 | 8.4 | 8.4 | 8.5 | 9.0 |
|  | Misc | 50.5 | 50.5 | 50.6 | 50.8 | 50.7 | 50.2 |
| 2 | Diab | 20.5 | 20.8 | 20.5 | 19.6 | 20.7 | 21.0 |
|  | Glom | 14.0 | 14.2 | 13.9 | 14.0 | 13.7 | 13.1 |
|  | Polyc | 6.5 | 6.5 | 6.5 | 6.5 | 6.5 | 7.2 |
|  | Pyelo | 8.5 | 8.7 | 8.8 | 8.7 | 8.9 | 9.3 |
|  | Other | 50.4 | 49.8 | 50.2 | 51.2 | 50.3 | 49.5 |
| 3 | Diab | 20.8 | 21.1 | 20.0 | 19.5 | 20.8 | 20.3 |
|  | Glom | 13.9 | 14.3 | 13.9 | 14.0 | 13.5 | 12.9 |
|  | Polyc | 6.6 | 6.7 | 6.8 | 6.5 | 6.6 | 7.7 |
|  | Pyelo | 8.4 | 8.3 | 8.3 | 8.8 | 8.4 | 8.6 |
|  | Other | 50.4 | 49.7 | 51.1 | 51.1 | 50.7 | 50.6 |
| 4 | Diab | 21.0 | 21.5 | 20.1 | 19.3 | 21.0 | 19.7 |
|  | Glom | 13.8 | 14.4 | 14.2 | 14.5 | 14.0 | 14.6 |
|  | Polyc | 6.6 | 6.5 | 7.0 | 6.5 | 6.5 | 6.9 |
|  | Pyelo | 8.2 | 8.2 | 8.3 | 8.5 | 7.7 | 8.2 |
|  | Other | 50.4 | 49.5 | 50.5 | 51.3 | 50.8 | 50.6 |
| 5 | Diab | 21.2 | 21.8 | 19.8 | 19.3 | 21.3 | 20.9 |
|  | Glom | 14.0 | 14.7 | 14.5 | 14.3 | 14.6 | 13.8 |
|  | Polyc | 6.7 | 6.5 | 7.4 | 7.0 | 6.5 | 7.2 |
|  | Pyelo | 8.2 | 8.2 | 7.9 | 8.5 | 7.7 | 8.0 |
|  | Other | 49.9 | 48.9 | 50.4 | 50.9 | 50.0 | 50.2 |
| 6 | Diab | 21.3 | 21.8 | 19.0 | 19.9 | 21.0 | 20.0 |
|  | Glom | 13.9 | 14.7 | 14.5 | 14.4 | 14.9 | 14.0 |
|  | Polyc | 6.6 | 6.4 | 7.4 | 6.7 | 6.2 | 6.5 |
|  | Pyelo | 8.1 | 8.3 | 8.7 | 8.2 | 8.0 | 9.0 |
|  | Other | 50.1 | 48.9 | 50.4 | 50.8 | 50.0 | 50.5 |
| 7 | Diab | 21.3 | 21.7 | 16.6 | 19.8 | 21.3 | 17.1 |
|  | Glom | 14.2 | 14.8 | 14.9 | 14.6 | 15.3 | 14.8 |
|  | Polyc | 6.4 | 6.2 | 7.7 | 6.8 | 6.2 | 5.9 |
|  | Pyelo | 8.1 | 8.4 | 8.7 | 8.3 | 7.8 | 8.8 |
|  | Other | 50.1 | 48.8 | 52.2 | 50.6 | 49.4 | 53.3 |
| 8 | Diab | 21.7 | 21.8 | 16.1 | 20.4 | 21.4 | 15.6 |
|  | Glom | 14.2 | 15.1 | 15.4 | 15.0 | 16.5 | 16.1 |
|  | Polyc | 6.4 | 6.2 | 8.8 | 6.6 | 6.1 | 7.4 |
|  | Pyelo | 8.1 | 8.3 | 8.4 | 8.2 | 7.5 | 8.0 |
|  | Other | 49.7 | 48.6 | 51.4 | 49.8 | 48.6 | 53.0 |

Notes: Abbreviations: Diab = diabetes; Glom = glomerulonephritis; Polyc = polycystic kidney disease; Pyelo = pyelonephritis; Other = other, uncertain, or missing primary kidney disease. This category includes patients with hypertension, renovascular disease, other renal diagnoses, uncertain primary kidney disease, or missing data.

**Table A5 Distribution of heart failure comorbidity status (%), before and after inverse probability weighting, by haemoglobin target strategy and follow-up month.**

|  | Heart |  | Unweighted |  |  | Weighted |  |
| --- | --- | --- | --- | --- | --- | --- | --- |
| Month | failure | 95–115 g/L | 105–125 g/L | 125–145 g/L | 95–115 g/L | 105–125 g/L | 125–145 g/L |
| 0 | No | 32.2 | 32.2 | 32.2 | 32.2 | 32.2 | 32.2 |
|  | Yes | 9.8 | 9.8 | 9.8 | 9.8 | 9.8 | 9.8 |
|  | Miss | 58.1 | 58.1 | 58.1 | 58.1 | 58.1 | 58.1 |
| 1 | No | 32.2 | 32.2 | 32.3 | 33.0 | 33.3 | 32.7 |
|  | Yes | 9.9 | 9.9 | 9.8 | 9.8 | 10.2 | 9.8 |
|  | Miss | 57.9 | 57.9 | 57.8 | 57.2 | 56.5 | 57.5 |
| 2 | No | 33.5 | 32.9 | 34.3 | 35.0 | 36.0 | 36.5 |
|  | Yes | 9.9 | 9.4 | 9.1 | 9.8 | 9.6 | 8.9 |
|  | Miss | 56.6 | 57.7 | 56.5 | 55.3 | 54.4 | 54.6 |
| 3 | No | 34.4 | 33.4 | 35.5 | 35.4 | 37.0 | 36.9 |
|  | Yes | 10.3 | 9.5 | 9.2 | 10.2 | 9.7 | 8.7 |
|  | Miss | 55.3 | 57.2 | 55.4 | 54.5 | 53.3 | 54.4 |
| 4 | No | 35.0 | 33.5 | 34.7 | 35.4 | 37.8 | 36.9 |
|  | Yes | 10.5 | 9.7 | 9.3 | 10.8 | 10.4 | 9.6 |
|  | Miss | 54.5 | 56.8 | 56.0 | 53.8 | 51.9 | 53.5 |
| 5 | No | 36.4 | 34.4 | 35.5 | 36.9 | 39.2 | 39.1 |
|  | Yes | 10.6 | 9.4 | 8.7 | 11.3 | 10.4 | 9.4 |
|  | Miss | 53.0 | 56.2 | 55.9 | 51.8 | 50.5 | 51.5 |
| 6 | No | 36.9 | 34.9 | 35.5 | 37.6 | 39.3 | 38.5 |
|  | Yes | 11.0 | 9.5 | 7.8 | 11.6 | 10.6 | 8.9 |
|  | Miss | 52.1 | 55.5 | 56.8 | 50.9 | 50.1 | 52.6 |
| 7 | No | 37.2 | 35.5 | 35.8 | 38.6 | 39.8 | 38.0 |
|  | Yes | 11.1 | 9.3 | 7.0 | 11.3 | 10.5 | 9.2 |
|  | Miss | 51.7 | 55.2 | 57.2 | 50.2 | 49.7 | 52.8 |
| 8 | No | 37.8 | 36.3 | 35.7 | 39.8 | 40.3 | 36.5 |
|  | Yes | 11.2 | 9.5 | 7.0 | 11.0 | 10.6 | 9.8 |
|  | Miss | 51.1 | 54.2 | 57.3 | 49.2 | 49.1 | 53.7 |

**Table A6 Distribution of time-updated ferritin (µg/L; categories %) before and after inverse probability weighting, by haemoglobin target strategy and follow-up month.**

|  |  |  | Unweighted |  |  | Weighted |  |
| --- | --- | --- | --- | --- | --- | --- | --- |
| Month | Ferririn | 95–115 g/L | 105–125 g/L | 125–145 g/L | 95–115 g/L | 105–125 g/L | 125–145 g/L |
| 0 | <300 | 34.5 | 34.5 | 34.5 | 34.5 | 34.5 | 34.5 |
|  | 300-449 | 18.2 | 18.2 | 18.2 | 18.2 | 18.2 | 18.2 |
|  | 500+ | 17.5 | 17.5 | 17.5 | 17.5 | 17.5 | 17.5 |
|  | Missing | 29.8 | 29.8 | 29.8 | 29.8 | 29.8 | 29.8 |
| 1 | <300 | 41.6 | 41.5 | 41.4 | 41.5 | 42.1 | 42.7 |
|  | 300-449 | 23.6 | 23.7 | 23.4 | 23.8 | 23.3 | 23.1 |
|  | 500+ | 22.2 | 22.3 | 22.3 | 22.2 | 21.9 | 21.6 |
|  | Missing | 12.6 | 12.6 | 12.8 | 12.5 | 12.7 | 12.7 |
| 2 | <300 | 45.1 | 44.8 | 43.6 | 44.4 | 44.8 | 44.7 |
|  | 300-449 | 26.4 | 26.8 | 26.6 | 26.1 | 25.7 | 26.4 |
|  | 500+ | 23.4 | 23.5 | 24.8 | 24.6 | 24.3 | 23.7 |
|  | Missing | 5.1 | 4.9 | 5.1 | 4.8 | 5.2 | 5.1 |
| 3 | <300 | 44.9 | 43.6 | 40.6 | 43.5 | 43.7 | 43.9 |
|  | 300-449 | 27.3 | 28.3 | 28.4 | 27.1 | 27.7 | 27.7 |
|  | 500+ | 24.7 | 25.3 | 28.2 | 26.7 | 25.8 | 25.6 |
|  | Missing | 3.1 | 2.9 | 2.9 | 2.7 | 2.8 | 2.8 |
| 4 | <300 | 43.4 | 41.7 | 38.8 | 41.8 | 43.1 | 42.7 |
|  | 300-449 | 27.8 | 29.1 | 29.0 | 28.4 | 28.1 | 26.8 |
|  | 500+ | 26.4 | 27.1 | 30.3 | 27.7 | 26.9 | 29.0 |
|  | Missing | 2.4 | 2.2 | 2.0 | 2.2 | 1.9 | 1.5 |
| 5 | <300 | 40.5 | 38.7 | 35.6 | 39.4 | 40.9 | 38.4 |
|  | 300-449 | 28.9 | 30.1 | 29.7 | 29.6 | 28.4 | 28.4 |
|  | 500+ | 28.2 | 29.2 | 32.9 | 29.4 | 29.0 | 31.9 |
|  | Missing | 2.4 | 2.0 | 1.8 | 1.6 | 1.8 | 1.3 |
| 6 | <300 | 37.0 | 34.5 | 32.9 | 36.7 | 37.3 | 36.4 |
|  | 300-449 | 30.5 | 32.6 | 30.8 | 30.3 | 30.9 | 29.7 |
|  | 500+ | 30.6 | 31.4 | 34.7 | 31.5 | 30.5 | 32.7 |
|  | Missing | 2.0 | 1.5 | 1.5 | 1.5 | 1.4 | 1.2 |
| 7 | <300 | 34.8 | 32.4 | 29.7 | 33.7 | 33.5 | 32.5 |
|  | 300-449 | 31.3 | 33.2 | 29.9 | 31.8 | 32.5 | 29.9 |
|  | 500+ | 32.6 | 33.3 | 38.8 | 33.4 | 33.1 | 36.1 |
|  | Missing | 1.4 | 1.2 | 1.5 | 1.2 | 1.0 | 1.4 |
| 8 | <300 | 33.6 | 31.2 | 30.1 | 31.8 | 32.8 | 31.5 |
|  | 300-449 | 30.7 | 31.8 | 28.1 | 30.6 | 30.1 | 28.3 |
|  | 500+ | 34.3 | 35.7 | 40.1 | 36.6 | 36.0 | 39.0 |
|  | Missing | 1.5 | 1.4 | 1.7 | 1.0 | 1.0 | 1.2 |

**Table A7 Distribution of time-updated serum albumin (g/L; categories %) before and after inverse probability weighting, by haemoglobin target strategy and follow-up month.**

|  |  |  | Unweighted |  |  | Weighted |  |
| --- | --- | --- | --- | --- | --- | --- | --- |
| Month | Albumin | 95–115 g/L | 105–125 g/L | 125–145 g/L | 95–115 g/L | 105–125 g/L | 125–145 g/L |
| 0 | <35 | 46.4 | 46.4 | 46.4 | 46.4 | 46.4 | 46.4 |
|  | 35-39 | 29.4 | 29.4 | 29.4 | 29.4 | 29.4 | 29.4 |
|  | 40+ | 20.6 | 20.6 | 20.6 | 20.6 | 20.6 | 20.6 |
|  | Missing | 3.7 | 3.7 | 3.7 | 3.7 | 3.7 | 3.7 |
| 1 | <35 | 42.2 | 42.2 | 42.8 | 43.2 | 43.5 | 41.4 |
|  | 35-39 | 33.6 | 33.6 | 33.6 | 33.3 | 33.2 | 33.7 |
|  | 40+ | 23.4 | 23.5 | 22.9 | 22.8 | 22.6 | 24.3 |
|  | Missing | 0.8 | 0.8 | 0.8 | 0.7 | 0.7 | 0.6 |
| 2 | <35 | 36.4 | 35.2 | 38.8 | 39.3 | 39.4 | 40.9 |
|  | 35-39 | 35.8 | 35.6 | 34.6 | 34.6 | 33.7 | 32.8 |
|  | 40+ | 27.2 | 28.7 | 26.1 | 25.7 | 26.4 | 26.0 |
|  | Missing | 0.6 | 0.5 | 0.5 | 0.5 | 0.6 | 0.4 |
| 3 | <35 | 34.6 | 32.4 | 36.1 | 36.5 | 37.9 | 39.5 |
|  | 35-39 | 35.4 | 35.8 | 35.5 | 34.6 | 33.1 | 33.6 |
|  | 40+ | 29.4 | 31.3 | 27.8 | 28.2 | 28.5 | 26.5 |
|  | Missing | 0.6 | 0.6 | 0.6 | 0.6 | 0.6 | 0.4 |
| 4 | <35 | 33.2 | 30.7 | 35.2 | 34.4 | 36.2 | 38.7 |
|  | 35-39 | 35.1 | 35.5 | 35.6 | 33.8 | 33.6 | 34.0 |
|  | 40+ | 30.9 | 33.2 | 28.4 | 30.8 | 29.4 | 26.6 |
|  | Missing | 0.8 | 0.7 | 0.8 | 0.9 | 0.8 | 0.7 |
| 5 | <35 | 33.0 | 29.8 | 32.5 | 33.5 | 34.4 | 36.6 |
|  | 35-39 | 33.7 | 35.0 | 35.3 | 33.3 | 33.0 | 33.0 |
|  | 40+ | 32.2 | 34.4 | 31.2 | 32.4 | 31.5 | 29.8 |
|  | Missing | 1.1 | 0.8 | 1.0 | 0.9 | 1.1 | 0.7 |
| 6 | <35 | 32.2 | 29.4 | 32.6 | 32.9 | 33.5 | 36.7 |
|  | 35-39 | 34.4 | 34.8 | 33.9 | 34.2 | 32.7 | 33.4 |
|  | 40+ | 32.3 | 34.9 | 32.7 | 32.0 | 33.0 | 29.3 |
|  | Missing | 1.2 | 0.9 | 0.8 | 1.0 | 0.8 | 0.6 |
| 7 | <35 | 32.3 | 29.5 | 30.2 | 32.4 | 33.6 | 35.1 |
|  | 35-39 | 33.7 | 34.6 | 35.6 | 34.0 | 33.3 | 35.1 |
|  | 40+ | 33.3 | 35.3 | 33.5 | 32.9 | 32.7 | 29.0 |
|  | Missing | 0.8 | 0.6 | 0.8 | 0.8 | 0.5 | 0.8 |
| 8 | <35 | 32.5 | 29.5 | 27.2 | 31.9 | 32.9 | 28.6 |
|  | 35-39 | 33.3 | 34.3 | 35.6 | 34.7 | 33.4 | 38.3 |
|  | 40+ | 33.2 | 35.3 | 36.0 | 32.6 | 32.9 | 32.0 |
|  | Missing | 1.0 | 0.9 | 1.2 | 0.8 | 0.8 | 1.0 |

**Table A8 Distribution of time-updated white blood count (10^9^/L; categories %) before and after inverse probability weighting, by haemoglobin target strategy and follow-up month.**

|  |  |  | Unweighted |  |  | Weighted |  |
| --- | --- | --- | --- | --- | --- | --- | --- |
| Month | WBC | 95–115 g/L | 105–125 g/L | 125–145 g/L | 95–115 g/L | 105–125 g/L | 125–145 g/L |
| 0 | <6 | 26.8 | 26.8 | 26.8 | 26.8 | 26.8 | 26.8 |
|  | 6-6.9 | 16.1 | 16.1 | 16.1 | 16.1 | 16.1 | 16.1 |
|  | 7-7.9 | 15.9 | 15.9 | 15.9 | 15.9 | 15.9 | 15.9 |
|  | 8-8.9 | 13.0 | 13.0 | 13.0 | 13.0 | 13.0 | 13.0 |
|  | 9+ | 28.3 | 28.3 | 28.3 | 28.3 | 28.3 | 28.3 |
| 1 | <6 | 27.0 | 26.9 | 27.1 | 26.8 | 26.6 | 26.7 |
|  | 6-6.9 | 17.5 | 17.5 | 17.4 | 17.4 | 17.3 | 17.3 |
|  | 7-7.9 | 16.0 | 16.1 | 16.1 | 16.0 | 16.3 | 16.2 |
|  | 8-8.9 | 12.9 | 12.8 | 12.8 | 12.8 | 12.8 | 12.9 |
|  | 9+ | 26.6 | 26.7 | 26.6 | 27.1 | 27.0 | 26.9 |
| 2 | <6 | 27.4 | 26.9 | 27.2 | 27.8 | 27.2 | 27.1 |
|  | 6-6.9 | 18.1 | 18.3 | 17.9 | 17.5 | 17.3 | 17.3 |
|  | 7-7.9 | 17.1 | 17.3 | 17.3 | 16.4 | 17.0 | 16.5 |
|  | 8-8.9 | 12.8 | 13.0 | 12.5 | 12.5 | 13.2 | 13.0 |
|  | 9+ | 24.6 | 24.6 | 25.2 | 25.8 | 25.3 | 26.1 |
| 3 | <6 | 27.5 | 27.0 | 27.8 | 28.3 | 27.3 | 27.0 |
|  | 6-6.9 | 18.3 | 18.4 | 17.9 | 17.8 | 17.4 | 18.3 |
|  | 7-7.9 | 17.3 | 17.5 | 16.3 | 16.8 | 16.9 | 16.1 |
|  | 8-8.9 | 13.4 | 13.5 | 12.9 | 12.6 | 13.4 | 13.8 |
|  | 9+ | 23.5 | 23.7 | 25.0 | 24.6 | 25.0 | 24.8 |
| 4 | <6 | 27.7 | 27.1 | 27.8 | 27.4 | 26.9 | 25.8 |
|  | 6-6.9 | 18.1 | 18.6 | 17.9 | 18.8 | 18.8 | 17.4 |
|  | 7-7.9 | 17.3 | 17.4 | 16.7 | 17.1 | 16.6 | 17.9 |
|  | 8-8.9 | 13.2 | 13.0 | 12.9 | 11.6 | 11.9 | 12.6 |
|  | 9+ | 23.8 | 23.9 | 24.7 | 25.2 | 25.8 | 26.3 |
| 5 | <6 | 27.0 | 26.6 | 26.5 | 26.3 | 25.9 | 24.1 |
|  | 6-6.9 | 18.6 | 18.7 | 18.3 | 19.0 | 19.3 | 20.2 |
|  | 7-7.9 | 16.6 | 17.4 | 15.8 | 16.5 | 16.2 | 14.7 |
|  | 8-8.9 | 13.7 | 13.8 | 14.5 | 13.1 | 13.3 | 15.9 |
|  | 9+ | 24.1 | 23.6 | 24.9 | 25.2 | 25.3 | 25.1 |
| 6 | <6 | 28.5 | 27.8 | 26.8 | 27.1 | 26.4 | 25.1 |
|  | 6-6.9 | 18.0 | 18.1 | 18.5 | 18.8 | 19.1 | 20.6 |
|  | 7-7.9 | 16.7 | 17.4 | 16.8 | 16.8 | 17.0 | 15.5 |
|  | 8-8.9 | 13.8 | 14.3 | 14.1 | 14.4 | 14.7 | 14.5 |
|  | 9+ | 23.0 | 22.4 | 23.8 | 22.9 | 22.8 | 24.4 |
| 7 | <6 | 28.1 | 27.2 | 25.4 | 28.5 | 26.6 | 24.0 |
|  | 6-6.9 | 19.3 | 19.3 | 19.8 | 18.3 | 17.7 | 18.7 |
|  | 7-7.9 | 17.7 | 17.4 | 16.3 | 17.8 | 18.8 | 16.9 |
|  | 8-8.9 | 12.4 | 13.3 | 13.7 | 13.2 | 13.7 | 14.0 |
|  | 9+ | 22.5 | 22.8 | 24.9 | 22.3 | 23.2 | 26.4 |
| 8 | <6 | 29.0 | 27.2 | 25.1 | 28.4 | 25.8 | 23.3 |
|  | 6-6.9 | 18.6 | 19.0 | 19.2 | 18.5 | 18.6 | 19.8 |
|  | 7-7.9 | 17.1 | 17.2 | 16.9 | 17.8 | 17.5 | 16.1 |
|  | 8-8.9 | 12.8 | 13.7 | 14.3 | 12.8 | 15.2 | 15.9 |
|  | 9+ | 22.5 | 22.9 | 24.6 | 22.6 | 23.0 | 24.8 |

**Table A9 Distribution of time-updated C-reactive protein (mg/L; categories %) before and after inverse probability weighting, by haemoglobin target strategy and follow-up month.**

|  |  |  | Unweighted |  |  | Weighted |  |
| --- | --- | --- | --- | --- | --- | --- | --- |
| Month | CRP | 95–115 g/L | 105–125 g/L | 125–145 g/L | 95–115 g/L | 105–125 g/L | 125–145 g/L |
| 0 | 0 / not tested | 49.3 | 49.3 | 49.3 | 49.3 | 49.3 | 49.3 |
|  | 0.1 to 4.9 | 5.1 | 5.1 | 5.1 | 5.1 | 5.1 | 5.1 |
|  | 5 to 19.9 | 22.4 | 22.4 | 22.4 | 22.4 | 22.4 | 22.4 |
|  | 20+ | 23.2 | 23.2 | 23.2 | 23.2 | 23.2 | 23.2 |
| 1 | 0 / not tested | 49.0 | 49.1 | 49.2 | 48.0 | 47.7 | 48.9 |
|  | 0.1 to 4.9 | 5.9 | 5.9 | 5.8 | 6.1 | 6.1 | 6.1 |
|  | 5 to 19.9 | 23.5 | 23.5 | 23.2 | 23.7 | 23.6 | 23.6 |
|  | 20+ | 21.6 | 21.6 | 21.8 | 22.3 | 22.6 | 21.5 |
| 2 | 0 / not tested | 48.6 | 49.8 | 49.8 | 46.7 | 46.6 | 48.1 |
|  | 0.1 to 4.9 | 6.1 | 6.3 | 5.8 | 6.5 | 6.4 | 6.2 |
|  | 5 to 19.9 | 25.2 | 25.0 | 24.0 | 25.0 | 25.4 | 24.5 |
|  | 20+ | 20.2 | 19.0 | 20.5 | 21.8 | 21.6 | 21.2 |
| 3 | 0 / not tested | 47.8 | 48.5 | 46.5 | 44.7 | 44.2 | 43.8 |
|  | 0.1 to 4.9 | 6.7 | 7.0 | 5.8 | 6.7 | 6.9 | 5.9 |
|  | 5 to 19.9 | 25.1 | 25.4 | 25.1 | 25.5 | 26.0 | 25.0 |
|  | 20+ | 20.4 | 19.0 | 22.6 | 23.1 | 23.0 | 25.3 |
| 4 | 0 / not tested | 46.3 | 47.0 | 44.2 | 42.4 | 41.0 | 41.3 |
|  | 0.1 to 4.9 | 7.1 | 7.4 | 6.6 | 7.3 | 7.5 | 6.6 |
|  | 5 to 19.9 | 26.9 | 27.3 | 27.5 | 28.6 | 28.7 | 27.6 |
|  | 20+ | 19.8 | 18.2 | 21.7 | 21.7 | 22.8 | 24.5 |
| 5 | 0 / not tested | 45.4 | 47.9 | 46.2 | 41.0 | 41.2 | 38.4 |
|  | 0.1 to 4.9 | 7.5 | 7.6 | 5.9 | 8.1 | 7.8 | 6.8 |
|  | 5 to 19.9 | 26.7 | 26.8 | 26.8 | 29.2 | 29.1 | 27.9 |
|  | 20+ | 20.4 | 17.8 | 21.2 | 21.7 | 22.0 | 26.9 |
| 6 | 0 / not tested | 44.8 | 46.9 | 46.7 | 40.3 | 40.6 | 38.1 |
|  | 0.1 to 4.9 | 7.6 | 7.8 | 5.4 | 8.0 | 7.5 | 7.7 |
|  | 5 to 19.9 | 27.3 | 27.6 | 28.3 | 29.5 | 30.4 | 27.1 |
|  | 20+ | 20.4 | 17.6 | 19.6 | 22.3 | 21.5 | 27.1 |
| 7 | 0 / not tested | 42.9 | 45.4 | 45.6 | 38.6 | 38.5 | 39.7 |
|  | 0.1 to 4.9 | 8.0 | 8.4 | 5.2 | 8.1 | 8.2 | 6.9 |
|  | 5 to 19.9 | 28.3 | 28.7 | 30.6 | 30.0 | 31.1 | 28.9 |
|  | 20+ | 20.8 | 17.5 | 18.6 | 23.4 | 22.2 | 24.5 |
| 8 | 0 / not tested | 42.7 | 45.9 | 45.4 | 37.7 | 39.2 | 41.2 |
|  | 0.1 to 4.9 | 8.1 | 8.0 | 4.3 | 8.4 | 8.5 | 4.2 |
|  | 5 to 19.9 | 28.7 | 28.7 | 33.0 | 31.0 | 31.2 | 33.5 |
|  | 20+ | 20.5 | 17.5 | 17.3 | 22.9 | 21.1 | 21.1 |
